# Simulating enzyme catalysis with electrostatically embedded machine learning potentials

**DOI:** 10.1039/d6sc01156j

**Published:** 2026-03-10

**Authors:** Valentin Gradisteanu, Elliot W. Chan, Lester Hedges, Meritxell Malagarriga, Rolf David, Miguel de la Puente, Damien Laage, Iñaki Tuñón, Marc W. van der Kamp, Kirill Zinovjev

**Affiliations:** a Departamento de Química Física, Universidad de Valencia 46100 Burjassot Spain kirill.zinovjev@uv.es; b School of Biochemistry & Cellular and Molecular Medicine, University of Bristol Biomedical Sciences Building, University Walk Bristol BS8 1TD UK marc.vanderkamp@bristol.ac.uk; c OpenBioSim Community Interest Company Edinburgh UK; d Laboratory CPCV, Department of Chemistry, École Normale Supérieure, PSL University, Sorbonne Université, CNRS Paris France

## Abstract

To simulate enzyme reactions, multiscale quantum mechanics/molecular mechanics (QM/MM) approaches are well established and popular. However, accurately and efficiently estimating enzyme activity is a challenge, because in general, precise methods are too computationally expensive. Here, we demonstrate that enzyme catalysis can be captured by coupling efficient, reactive machine-learned potentials (MLPs) trained on gas phase data to the wider enzyme environment using electrostatic machine learning embedding (EMLE). The EMLE scheme is first applied to the natural Diels–Alderase AbyU, showing that it correctly differentiates the catalytic action on different enzyme–substrate conformations. Then, we show that training a reaction-specific EMLE model allows us to accurately capture the enzyme catalytic effects of the conversion of chorismate to prephenate, a reaction with a highly polarizable and charged transition state. In both cases, in contrast to mechanical embedding approaches, the EMLE scheme allows accurate and efficient predictions of enzyme catalysis, agreeing with high-level QM/MM reference calculations. This approach facilitates the use of gas phase-trained MLPs in MLP/molecular mechanics (ML/MM) simulations and should thus be highly beneficial for computational activity screening of enzyme biocatalysts.

## Abstract 2

Enzyme catalysis can be simulated accurately and efficiently by coupling machine-learned potentials trained on gas-phase data to the environment (ML/MM) using electrostatic machine-learning embedding (EMLE).

## Graphical abstract

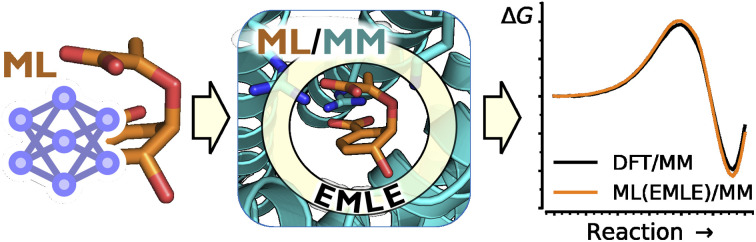

## Introduction

Computational methods that accurately and efficiently simulate reactions in condensed environments, such as solutions, interfaces or enzymatic active sites, are essential for the design of better catalysts and inhibitors for these processes. In the case of enzymes, such methods can be incorporated in computational workflows for optimizing covalent drugs, analysing drug breakdown (*e.g.* in drug metabolism and antimicrobial resistance) and designing (*de novo*) enzymes. The revolution in protein design aided by artificial intelligence in the past few years^1^ has recently been expanded to enzyme design, such that, for the first time, *de novo* enzymes with appreciable activity can be obtained directly through computational design.^2^ However, only a certain fraction (∼5–10%) of designed enzymes typically shows activity. Modern workflows combine protein scaffold design and optimization, sequence optimization and (all atom) structure prediction techniques to perform fully computational (re)design of efficient enzymes.^3,4^ To improve upon success rates for enzyme activity routinely, and to allow reaching catalytic efficiencies of native enzymes, these workflows typically still require extensive experimental screening, iterative computational-experimental design optimization, or rounds of lab-based directed evolution. To reduce the time and resources required for obtaining effective novel biocatalysts, efficient methods are needed to accurately predict enzyme catalysis from structure.

Quantum Mechanics/Molecular Mechanics (QM/MM) methods have played a key role in understanding enzymatic catalysis, providing a framework to obtain activation free energies and, in combination with Transition State Theory, to reproduce and predict enzymatic rate constants.^5–7^ QM/MM calculations have offered fundamental insights into reaction mechanisms, revealing the role of enzymatic active sites in stabilizing transition states and how the enzyme environment can lower reaction barriers, typically through electrostatic interactions.^8,9^ QM/MM simulations have thereby contributed substantially to understanding how enzymes work, establishing the origin of enzymatic catalysis,^10^ and can also provide good estimations of relative enzyme activities.^11,12^ However, despite their success, the quantitative use of QM/MM simulations remains constrained by the dual requirement of accuracy and sampling. On the one hand, a sufficiently accurate, high-level QM method is typically required to correctly describe bond-breaking and bond-forming processes, ensuring reliable energetics.^13^ On the other hand, extensive sampling is required to capture the conformational dynamics of the enzyme–substrate complex and its influence on the reaction coordinate.^14,15^ Satisfying both requirements simultaneously leads to extremely computationally expensive simulations, often limiting their application to a handful of enzyme variants at most. This challenge becomes particularly critical in biocatalyst and (covalent) drug design, where rapid and accurate predictions are needed to guide engineering efforts. Addressing this limitation requires alternative approaches that maintain high accuracy while drastically reducing computational cost.

The need to sacrifice the model precision to obtain better sampling can be circumvented by employing Machine Learning Potentials (MLPs).^16^ The promise of MLPs is to provide molecular energies and forces close to the level of theory used to train the potential, while being orders of magnitude faster. In the last decade, a plethora of approaches for developing reactive MLPs have emerged, including several open-source packages that facilitate MLP training and inference.^17–20^ Nevertheless, the computational cost of MLPs is still significantly larger than that of empirical MM force fields.^21^ Therefore, hybrid “ML/MM” potentials that apply an MLP only to the reactive region of the system would provide significant reduction of computational cost compared to both “full ML” and accurate QM/MM simulations, as well as being able to take advantage of established and extensively tested MM force fields.

In QM/MM simulations, the response of the QM region to the MM environment can be trivially described either through mechanical embedding (by assigning partial charges to the QM atoms based on gas phase electronic density) or more precisely using an electrostatic embedding (by allowing the electronic density to respond to the presence of the MM point charges). The majority of ML potentials only predict the energy and forces of a specific molecular system and ignore details about the electronic distribution. Such MLPs are therefore not capable of predicting the response to the environment. This issue was addressed by development of ML/MM coupling schemes of different complexity.^22^ The simplest solution to employ an MLP in the context of a hybrid ML/MM simulation is to treat the interaction between the ML and MM subsystems at the MM level of theory (MM embedding), by assigning fixed point charges to the ML atoms.^23–25^ While this approach was shown to be useful to improve calculations not involving chemical transformations, such as the prediction of protein–ligand binding free energies,^25^ it has limited applicability to reactive systems, where fixed MM charges are incapable of capturing the electronic rearrangements happening during the reaction. In this case, the description must be extended to include the varying interaction between the ML and MM regions. One way to do so is to provide an explicit representation of the MM environment to the ML model^26–30^ and learn the QM/MM energy directly. Such methods provide an MLP that can be used in combination with a specific MM environment or similar environments, such as point mutations in the enzymatic active site.^30^ A more general approach is to decompose the total QM/MM energy into the gas phase energy of the ML region (independent of the MM environment) and the embedding energy.^31–33^ Such a decomposition allows one to employ MLPs trained on solely gas phase reference data in ML/MM simulations. An important benefit of this is that, in principle, the effect of different environments (solution, enzyme variants) on a reaction modelled with the same MLP can be captured. This decomposition approach is used in our “electrostatic machine learning embedding” (EMLE) scheme, which uses a physically motivated ML architecture to learn the embedding energy. The EMLE scheme has already been shown to feature a high degree of transferability: a single model trained on QM calculations of a chemically diverse set of small molecules provides precise embedding energies for a number of ML subsystems and MM environments.^31,34,35^ Multiple methods based on a similar decomposition approach were recently proposed and applied to a range of problems, such as excited state molecular dynamics,^36^ protein–ligand interactions,^33^ solvent effects on reactivity,^37^ and response to an external electric field.^38^

**Fig. 1:**
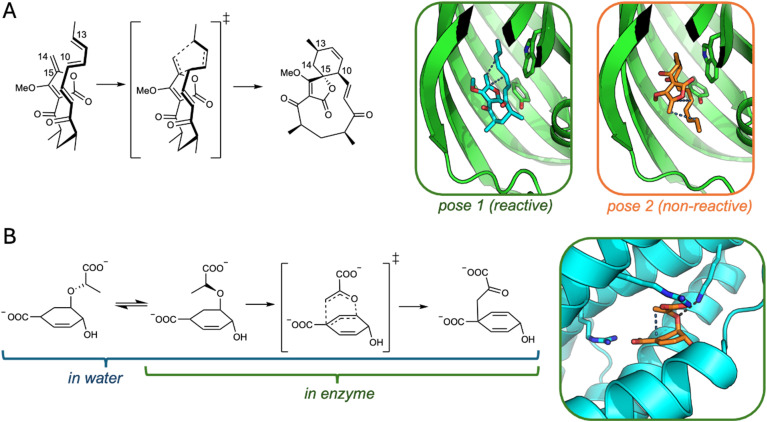
Reactions and enzyme–substrate poses investigated in this work. (A) Diels–Alder reaction catalysed by AbyU (*O*-methylated analogue of natural substrate). Carbon atoms involved in C–C bond formation are labelled. Two enzyme–substrate poses are shown (substrate and key residues W124 and Y76 as sticks, hydrogen atoms omitted), with bonds formed indicated by blue dashed lines. (B) Claisen rearrangement of chorismate to prephenate. The conformational equilibrium for chorismate (not sampled when bound to the enzyme) is also depicted. Enzyme–substrate pose shown indicates the C–C bond formed (blue dashed line), Shown as sticks are residues R25 and R101 involved in substrate binding, and the key catalytic K36 with its transition-state stabilizing interaction with the substrate ether oxygen (black dashed line).

The central message of this work is that by using our EMLE approach, an ML model can be trained exclusively on gas-phase reference QM data, whilst ensuring transferability of the embedding scheme to different MM environments. We demonstrate this by evaluating the performance of the EMLE scheme on two different reactions promoted by specific enzymes (Fig. 1): a natural Diels–Alder addition catalysed by the spirotetronate cyclase AbyU and the conversion of chorismate to prephenate catalysed by chorismate mutase (ChoM). Both enzymatic reactions are compared to the uncatalyzed process in water using the same ML/MM potential. These two systems present different challenges common in computational studies of enzymatic catalysis. In the case of AbyU, we show that accounting for the influence of the enzyme environment on the electronic distribution in the substrate is essential to predict relative activity of different enzyme–substrate conformations. In ChoM, we show that not only the charge distribution itself, but also the electronic response to the environment varies during the reaction, presenting an additional challenge to the embedding model. We show that the EMLE scheme successfully addresses both issues, resulting in good agreement between DFT/MM and ML/MM enzyme catalytic effects. Moreover, we show that the ChoM model can be directly applied to two variants of a distinct natural enzyme belonging to a different structural family, and correctly reproduces the difference in activation free energies upon mutation. To present our findings, we first provide a brief description of the EMLE scheme used. Then, we describe the protocols used for each enzyme system to obtain the reference data and train the bespoke MLPs and (in the case of ChoM) EMLE models. Notably, we deliberately train and use two conceptually different MLPs for the two enzyme reactions, to demonstrate the general applicability of the EMLE scheme (agnostic of the MLP approach used). In presenting the results, we analyse and discuss which aspects of the MLP embedding are essential to correctly describe reactivity in the condensed phases. Overall, we show how ML/MM simulations with EMLE-embedding can be used to provide an accurate, general description of the influence of the environment on chemical reactions, facilitating access to fast and accurate simulations of chemical processes in complex environments.

## Methods

### ML/MM embedding scheme

In this work we employ the EMLE embedding scheme^31^ implemented in the *emle-engine* package.^34^ Full details about the scheme and the implementation can be found in the corresponding publications. Briefly, in EMLE the total energy of an ML/MM system is decomposed in four terms, as follows:

1 *E*_ML/MM_ = *E*_gas_(***R***_ML_) + *E*_static_(***R***_ML_, ***R***_MM_) + *E*_induced_(***R***_ML_, ***R***_MM_) + *E*_MM_(***R***_ML_, ***R***_MM_)

where *E*_gas_ is the gas phase energy of the ML subsystem, *E*_static_ is the interaction energy between the unperturbed ML region and MM environment, *E*_induced_ is the induction energy, including the cost of polarizing the ML region by the MM field and *E*_MM_ is the MM energy, which includes the van der Waals and short-range repulsion interactions between ML and MM subsystems, which are treated at the MM level.

This decomposition offers multiple advantages. First, the explicit *E*_gas_ term allows to employ ML potentials trained on gas phase QM calculations (*i.e.* not explicitly trained for use in ML/MM simulations), provided that the MLP is valid for the conformations visited in condensed phase simulation. Second, the MM part is represented solely by charges and positions of the MM atoms, allowing to apply the scheme as a drop-in replacement of a QM backend in existing QM/MM codes. Third, separation of gas phase, static and induced components allows to divide the problem of training an electrostatically embedded ML potential into three simpler ones, monitoring the performance of each component separately by comparing to the reference QM calculations. *E*_gas_ corresponds to the total energy of the ML region *in vacuo*:

2 *E*_gas_ = 〈*Ψ*_0_|*Ĥ*_0_|*Ψ*_0_〉

*E*_static_ is the interaction between the unperturbed QM system and MM point charges, which can be estimated from the electrostatic potential of the unperturbed QM system *V*^el^_0_ at the MM atom positions:3
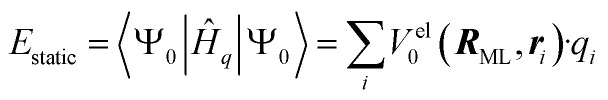
where ***r***_*i*_ and *q*_*i*_ are positions and charges of MM atoms, respectively. Finally, *E*_induced_ is the energy change upon allowing the point charges to polarize the ML region. In case of QM/MM, this component can be estimated as the difference between the full QM/MM energy and the sum of gas and static components:

4 *E*_induced_ = *E*_QM/MM_ − (*E*_gas_ + *E*_static_) = 〈*Ψ*|*Ĥ*|*Ψ*〉 − 〈*Ψ*_0_|*Ĥ*|*Ψ*_0_〉

where *Ĥ* = *Ĥ*_0_ + *Ĥ*_q_. In EMLE scheme, the induction term is obtained indirectly by learning gas phase molecular dipolar polarizability tensors, thus avoiding the need for explicit QM/MM energies.

The EMLE decomposition allows using different training approaches for each of the components. *E*_gas_ can be provided by any MLP architecture. In this work, this is showcased by using either a MACE^19^ or a DeePMD^18^ potential for the reactions studied here. As follows from eqn (3), *E*_static_ relies on accurate prediction of the electrostatic potential of the ML region, which in turn can be estimated by predicting the electronic density. In principle, any density prediction approach can be used in EMLE. The approach used here, as in previous applications of EMLE, is to predict the electronic density based on the MBIS decomposition,^39^ which describes the total density of the system as the sum of atomic contributions, approximated as spherical Slater densities. Higher order atomic multipoles (dipoles, quadrupoles, *etc.*) are ignored. Finally, *E*_induced_ is predicted by the Thole model^40^ which is based on the concept of atomic polarizability^41^ – each atom is treated as an isotropic polarizable center and responds to both the external field (generated by MM point charges) and the fields generated by the dipoles induced on the rest of the atoms. The key property to be predicted is therefore the atomic polarizability, which can be approximated as being proportional to the atomic volume:

5 *α* = *k*_*Z*_·*v*

Here, *k*_*Z*_ is a polarizability/volume (*α*/*v*) ratio defined per chemical element (as a learnable parameter) and the volume *v* can be obtained from the MBIS atomic Slater density.^31^ While the Thole model has been shown to struggle with long conjugated systems^42^ or high degrees of charge transfer,^43^ we expect it to perform well for the majority of enzymatic reactions. Moreover, by allowing for flexible *α*/*v* ratios, the predictions by the Thole model for highly polarizable transition state regions can be improved significantly, as shown below.

A further advantage of the decomposition presented in eqn (1) is that the importance of the individual terms for the studied process can be easily tested by modifying or turning off the components of the scheme. For instance, by removing the induced component (leading to what is commonly known as “Mechanical embedding”), the impact of polarization of the QM region can be estimated. Furthermore, replacing the static component by a simple Coulomb interaction between MM point charges and similar fixed partial charges assigned to QM atoms (“MM embedding”) allows to quantify the impact of electronic rearrangement. In this work we evaluate the performance of these three embedding variants, with the aim of serving as a guide for the development of future ML/MM simulations.

### Software and implementation

All the QM calculations presented in this work (for reference DFT/MM MD simulations and data for ML training) were performed with the *ORCA* 5.0.3 quantum chemistry package.^44^ The MBIS decomposition of electronic density was performed using *Horton* 2.3.0.^45^ EMLE model training, ML(EMLE)/MM MD simulations and error analysis in this work were performed using our recently developed *emle-engine* package.^34^*emle-engine* consists of three key components:

*emle-train*: a tool for training of bespoke EMLE models based on gas-phase QM calculations. Provides automatic parsing of *ORCA* outputs.

*emle-server*: an ML/MM backend that interfaces with MD engines. In this work, *emle-server* was interfaced with sander by reusing the *sander-ORCA* interface. This avoids the need for modifications in the sander source code and ensures direct comparability with QM/MM results obtained using *sander* + *ORCA*.

*emle-analyze*: a tool that calculates the energy components defined in eqn (2)–(4) for provided structures with ML(EMLE) and QM potentials. The reference QM values are obtained by parsing *ORCA* outputs for single point calculations of the QM region in gas phase and in the presence of MM point charges. The electrostatic potential at MM atomic positions (eqn (3)) required to calculate QM/MM *E*_static_ values was obtained with the *orca_vpot* tool.

Installation instructions and documentation for the *emle-engine* package can be found on the project website (https://chemle.github.io/emle-engine/).

### AbyU Diels–Alderase reaction

#### System preparation

For AbyU, simulation systems were taken from a previous study that considered different binding poses based on molecular docking of a close analogue of the natural product (*O*-methylated) in the AbyU crystal structure (PDB ID: 5DYV, chain A).^46^ Here, we used what were found to be the most and least reactive binding poses from this work, in order to see the effect of different embedding approaches on the relative barriers obtained with ML/MM. For each pose, the free energy profile or Potential of Mean Force (PMF) was obtained using umbrella sampling (US) along the previously optimized reaction coordinate (RC): 0.3 × *d*(C10–C15) + 0.7 × *d*(C13–C14).^46^ Sampling was performed between RC = 1.3 Å (product) and RC = 3.8 Å (substrate in enzyme-bound conformation), with 5 different replicas for each window based on starting structures from the previous QM(DFTB2)/MM US sampling.^46^ The same was done for the reaction simulation in water, based on equivalent QM(DFTB2)/MM US sampling.^47^ In each case, a single restraint (one-sided harmonic) was used to prevent formation of an unphysical chemical bond between C2 and C8 (force constant of 100 kcal mol^−1^ Å^−2^ when *d*(C2–C8) < 2.7 Å).

#### Reference DFT/MM calculations

Reference DFT/MM US simulations were performed using 26 windows (from 1.3 to 3.8 Å, step size 0.1 Å) with a RC restraint of 200 kcal mol^−1^ Å^−2^, as used before for DFTB2/MM simulations.^46^ This protocol was used both in enzyme and in water. 5 × 2 ps of DFT/MM MD simulations were performed for each window (timestep 1 fs), using the M06-2X functional^48^ (which performs well for Diels–Alder reactions^49^) and the 6-31G* basis set. The Grossfield implementation of WHAM was employed for PMF calculation.^50^

#### ML model training

As was shown previously, MLPs can be trained for basic Diels–Alder reactions, achieving chemical accuracy using data-efficient approaches.^51^ Here, we use a similar approach to train a specific MACE reactive MLP for the intra-molecular Diels–Alder reaction catalysed by AbyU using the *mlp-train* package^51^ with default settings, which automatically generates initial configurations based on a given transition state (TS) structure (here 170). This training set was supplemented with 20 structures from each window from the reference DFT/MM simulations (1 structure every 50 fs) to ensure the training set covered a range of geometries along the reaction pathway. The training set thus consisted of 690 structures (170 + 26 × 20). ML/MM US simulations were then performed with this initial MLP, 7 windows of which entered unphysical conformations (where just one of the two carbon–carbon bonds was formed). 2 structures from each of these windows were added to the training set, which was then used to train the final MLP (704 structures). Although this training step required system-specific human intervention, the overall computational cost is very low (only 704 DFT calculations required). Further, we anticipate that in the future, more general automated approaches can be used, or indeed (more) generic reactive MLPs will be developed that can be used together with our EMLE model directly. Subsequent ML/MM US simulations were performed using the same procedure as for the reference DFT/MM simulations, but now with 5 × 10 ps sampling per window.

### Chorismate mutase reaction

#### System preparation

The simulation system for the reaction in water solution was prepared by solvating the chorismate molecule in a box of 2614 TIP3P water molecules and 2 Na^+^ counterions, using the *antechamber* and *tleap* codes from the *AmberTools23* package.^52^ For *E. coli* ChoM (*Ec*ChoM), the starting structure (protein, substrate and solvent) was taken from previous work^53^ (based on PDB ID 1ECM), and reparameterized using ff19SB for the protein, TIP3P water, and GAFF2 (with AM1-BCC charges, assigned using *Antechamber*) for the substrate. Both systems were equilibrated with 200 ps of NPT MD at 300 K (Langevin thermostat) and 1 bar (Berendsen barostat) to relax the box size and subsequent 50 ns of NVT relaxation. A 2 fs timestep was used with SHAKE^54^ applied to constrain bonds involving hydrogens. All simulations were performed using *pmemd* from *Amber22* and *sander* from the *AmberTools23* package.^52^ For *B. subtilis* ChoM (*Bs*ChoM), the starting structure (protein and substrate) was taken from md1.rst from the CCPBioSim QM/MM workshop material^55^ (based on PDB ID: 2CHT), resolvated in a box of TIP3P solvent (minimal distance between protein and box edge of 12 Å) with 11 Na^+^ counterions to neutralize and parameterized as above (ff19SB, GAFF2). The same protocol as above was then used to equilibrate the simulation systems for *Bs*ChoM and its Arg90Cit variant, where the catalytically active Arg90 residue was mutated to the nonnatural residue citrulline (Cit), isosteric to arginine but neutral. Citrulline parameters were obtained using standard protocol of Antechamber, with RESP charges based on the HF/6-31G* density obtained with Gaussian16.^56^ After introducing the Arg90Cit mutation, the system was additionally equilibrated by 50 ns of NVT MD before switching to the ML/MM US protocol described below.

#### Reference DFT/MM calculations

The chorismate to prephenate reaction can be described accurately by a reaction coordinate (RC) defined as the difference between the length of the C–O bond that is breaking, and the length of the C–C bond that is forming (see Fig. 1). The reference DFT/MM PMFs were obtained using 68 (sampling RC from −4.5 Å to 2.2 Å, step size 0.1 Å) and 48 (sampling RC from −2.5 Å to 2.2 Å) US windows for the water and enzyme reactions, respectively. In each window a harmonic biasing potential with the force constant of 100 kcal mol^−1^ Å^−2^ was applied. The reaction in water spans a larger RC range because of the conformational change between the pseudo-diaxial and pseudo-diequatorial conformations of the reactants (see Fig. 1). This conformational change is not present in the enzyme reaction because the reactive pseudo-diequatorial conformation is already stabilized in the Michaelis complex.^57^ The initial structures for each US window were relaxed using DFTB3/MM MD (1 fs timestep) with the same RC biasing potential. Additionally, during relaxation several restraints were added to ensure no other chemical bonds were broken or formed. Then, 50 steps of DFT/MM MD were performed using B3LYP-D3BJ^58–60^ functional and 6-31G(d) basis set. Finally, 5 ps of production MD were performed for each window at the same level of theory. The PMFs were integrated using WHAM.^50^

#### ML model training

The initial training set was obtained from the short 50 step DFT/MM relaxation trajectories performed for reference DFT/MM US (see above) by removing the MM environment, keeping only the QM region (24 atoms). Structures for both water and enzyme simulations were combined to train a single model suitable for both environments. From each trajectory, 5 snapshots were taken every 10 steps, resulting in 116 × 5 = 580 structures. The single point energy and gradient estimates at the B3LYP-D3BJ/6-31G(d) level (same as in PMFs) were performed with *ORCA* 5.0.3.^44^ For bespoke EMLE model training, calculation of atomic polarizabilities and MBIS decomposition of electronic density were performed.

To demonstrate that our *emle-engine* framework is agnostic to the choice of specific MLP architecture, the gas phase potential for the ChoM reaction was obtained using DeePMD.^18^ Further, a bespoke EMLE model was trained for the chorismate to prephenate reaction. This was necessary, because the current generic EMLE model is trained on a dataset of neutral compounds only and is thus unable to describe the chorismate molecule that has a charge of −2. Both DeePMD and EMLE models for the chorismate transformation were trained by applying an active learning protocol facilitated by *ArcaNN* scripts:^61^ the models trained on the initial training set were used to propagate a series of ML/MM US trajectories and new structures were extracted to augment the training set. EMLE requires only *in vacuo* reference calculations, therefore we were able to use the same structures for training both models. Moreover, the training process as implemented in *emle-engine* is significantly faster for EMLE compared to DeePMD MLP, making the overhead of bespoke EMLE model training almost negligible compared to training only a DeePMD model.

The transition state of chorismate conversion to prephenate features highly polarizable oxyanion-like species, for which the simple atomic polarizability approximation based solely on atomic volume breaks down. Therefore, to improve EMLE performance, we adjusted the expression for the atomic polarizability to depend explicitly on the local environment by introducing an additional correction term:6
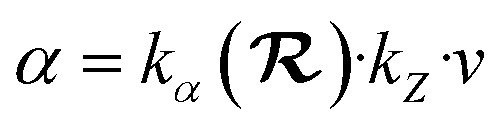
where *k*_*α*_ depends on the local atomic environment 
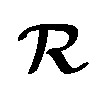
 (excluding the MM region) and is predicted with sparse Gaussian Process Regression in the same way as valence shell widths and electronegativities.^31^ The definition of *k*_*α*_ as a correction factor to the original, volume-based polarizability model allows one to easily introduce a regularization term during the model training, which biases *k*_*α*_ to stay close to unity. We observed that without such regularization the training procedure tends to overfit the model and perform poorly in subsequent ML/MM MD. Keeping *k*_*α*_ close to unity does not significantly impact the model training error while providing stable atomic polarizabilities during the MD simulations. Below, we refer to the polarizability model in eqn (5) as “fixed” and that defined in eqn (6) as “flexible”.

## Results

### Capturing differential enzyme catalysis: Diels–Alder reaction catalysed by AbyU

**Fig. 2:**
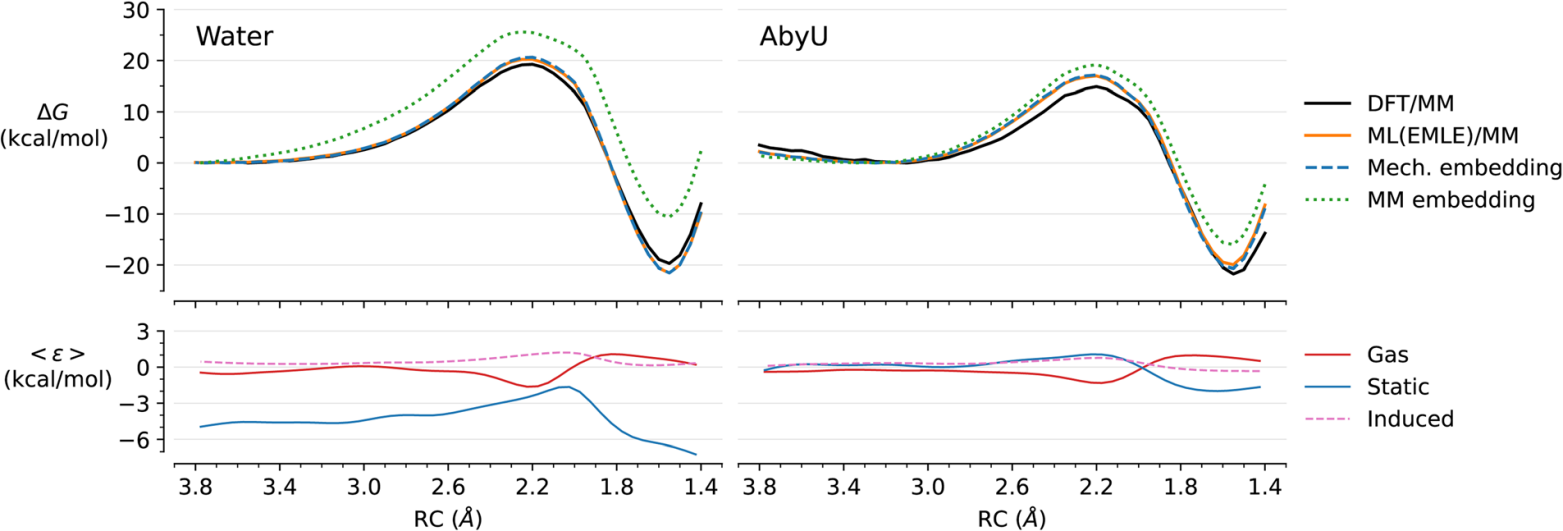
ML/MM simulations of the Diels–Alder reaction for the AbyU substrate in water and catalysed by AbyU. (Top) Free energy profiles obtained by umbrella sampling for the DFT/MM reference and ML/MM with different embedding schemes. All ML/MM profiles use the same MACE MLP. (Bottom) Mean error of the ML(EMLE)/MM potential components in each window. The errors are interpolated using smooth cubic splines interpolation for clarity. Raw errors (without interpolation) are provided in Fig. S1.

To determine free energy profiles, potentials of mean force (PMFs) for the Diels–Alder reaction catalysed by the spirotetronate Diels–Alderase AbyU were obtained in the enzyme and in water. This reaction features a transition state that reflects the asynchronous but concerted nature of the reaction both in gas phase and in the enzyme, with the C13–C14 bond more advanced than C10–C15.^62^ The transition state is not highly polarized, and the main electronic effects promoting the reaction are internal to the substrate (*e.g.* dienophile activated by the electron withdrawing effect of the tetronate moiety). However, the (permanent) electric field provided by the enzyme was shown to be catalytic, for the reactive pose.^47^ The DFT/MM and ML(EMLE)/MM free energy profiles are in good agreement both in water and in enzyme (Fig. 2 and Table S1). The differences between ML/MM and DFT/MM can be ascribed to a combination of a lack of full convergence of the DFT/MM PMF (compared to ML(EMLE)/MM, Table S2), small errors in the ML(EMLE) potential, and statistical error. Notably, ML/MM with mechanical embedding results in almost identical PMFs as with the full EMLE embedding. This is expected here, due to the minimal difference in substrate's polarization along the Diels–Alder reaction progress. However, when using (fixed point-charge) MM embedding, the agreement is noticeably worse, both in water and in enzyme. Here, the expected changes in the electronic density along the reaction is not reflected by a change in the atomic charges, thus leading to relatively poor agreement with the reference DFT/MM PMFs. Hence, even in systems that are not highly polarized, it is still imperative to capture the response of the ML region to its electrostatic environment to obtain a reasonable free energy description of the process.

By construction, the EMLE scheme allows independent analysis of the errors for each of the three energy components defined in eqn (2)–(4). Therefore, to further analyze the performance of the EMLE scheme in context of this reaction, the difference between DFT/MM and ML(EMLE)/MM was decomposed into these three separate components by comparison to single-point DFT calculations (using *emle-analyze*, see Methods). This thus reflects the errors in the MLP itself (gas phase energy), the static component of EMLE energy, and the induced component of EMLE (Fig. 2, bottom). The intrinsic error in the MACE MLP is low, though it is noted that the largest errors occur around the TS (−2.0 kcal mol^−1^ in water, −1.5 kcal mol^−1^ in the enzyme). The MLP performs particularly well for the broad reactant minimum, which had the greatest number of structures in the training set. The MLP was obtained with just one iteration of providing additional structures, in a data- and computationally efficient approach (requiring only 704 DFT calculations). The intrinsic MACE MLP error can be reduced to below 1.0 kcal mol^−1^ along the reaction in both environments by slightly increasing the number of structures included between the TS region and the product (see Fig. S1). However, the current MLP is already sufficiently accurate for our purposes: demonstration of how the generic EMLE model captures key catalytic effects in the AbyU reaction. In both systems, the error in the induced component is low (close to zero, with maxima <2 kcal mol^−1^), which was expected given the low change in polarization along the reaction (which also explains the good performance of mechanical embedding). For the reaction in water, the static EMLE error is the largest contribution to the total error in the energies, whereas this is not the case for the reaction in the enzyme. This may be due to a combination of errors in the generic EMLE model used here,^31^ which was not optimized for this specific reaction, and the fact that the substrate has substantially larger conformational freedom in solution, with likely frequent changes in water-substrate hydrogen bonding interactions. In contrast, in the enzyme system, the substrate is enclosed in a tighter cavity, where interactions are essentially constant. Overall, however, the accuracy of the PMFs generated, along with the small errors for the enzyme system (most relevant for enzyme-catalysed reactions), suggests that the generic EMLE model is already sufficiently accurate for use in enzyme reaction simulations of charge neutral systems with low polarizability.

**Fig. 3:**
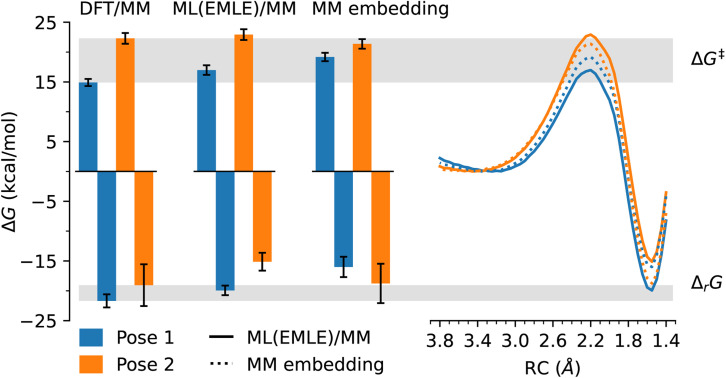
Differences between free energy barriers and reaction energies of the AbyU Diels–Alder reaction for two different AbyU-substrate poses. (Left) Free energy barriers (positive) and reaction free energies (negative) for both AbyU-poses as extracted from free energy profiles of the reference DFT/MM (differences between poses indicated as grey horizontal bars) and the ML/MM simulations. The errors indicate standard deviations obtained from PMFs calculated on US windows from each of the 5 individual replicas (see Methods). (Right) Free energy profiles of the reaction in AbyU for both poses with ML(EMLE)/MM (solid lines) and ML/MM with MM embedding (dashed lines).

To test the importance of accurate embedding (that includes the conformational dependence of the electronic distribution) in ML/MM simulations for capturing differences in reactivity, we performed ML(EMLE)/MM simulations with an additional substrate binding pose (Pose 2, see Fig. 1). This pose was previously found to be unreactive (based on semi-empirical QM/MM simulations^46^) due to the anticatalytic orientation of the enzyme electric field in this case.^47^ DFT/MM PMFs obtained here indeed show the activation energy for the unreactive pose (Pose 2) to be ∼7 kcal mol^−1^ higher than for the true Michaelis complex (Pose 1), in agreement with the previous semi-empirical QM/MM results. ML/(EMLE)/MM simulations capture this difference in activation energies accurately, whilst the difference is almost eliminated with MM embedding (Fig. 3). It is clear that the specific catalytic effect of the enzyme for the reactive pose, and also the difference in reactivity for alternative substrate poses, cannot be captured using MM embedding. It can be expected, as discussed below, that similar shortcomings may also apply to mechanical embedding for enzyme reactions with larger differential transition state stabilizations.

### Reaction with highly polarized transition state: chorismate mutase

Accurate embedding of the chorismate conversion to prephenate is more challenging than for the AbyU Diels–Alder reaction, due to large changes in the electronic structure and polarizability during the reaction process. The electron density on the ether oxygen O involved in bond breaking increases significantly when approaching the TS, stabilized by the active site interactions with the terminal NH_3_^+^ group of Lys36 and the backbone amide of Glu246, followed by reduction of the partial charge as it becomes the carbonyl oxygen of prephenate. These changes imply differences in both static and induced interactions between the substrate and the environment that must be accurately described by the embedding model. Moreover, the impact of this electronic rearrangement on the free energy profile of the reaction should depend strongly on the environment: electrostatic stabilization of the transient oxyanion reduces the activation free energy.^63^

**Fig. 4:**
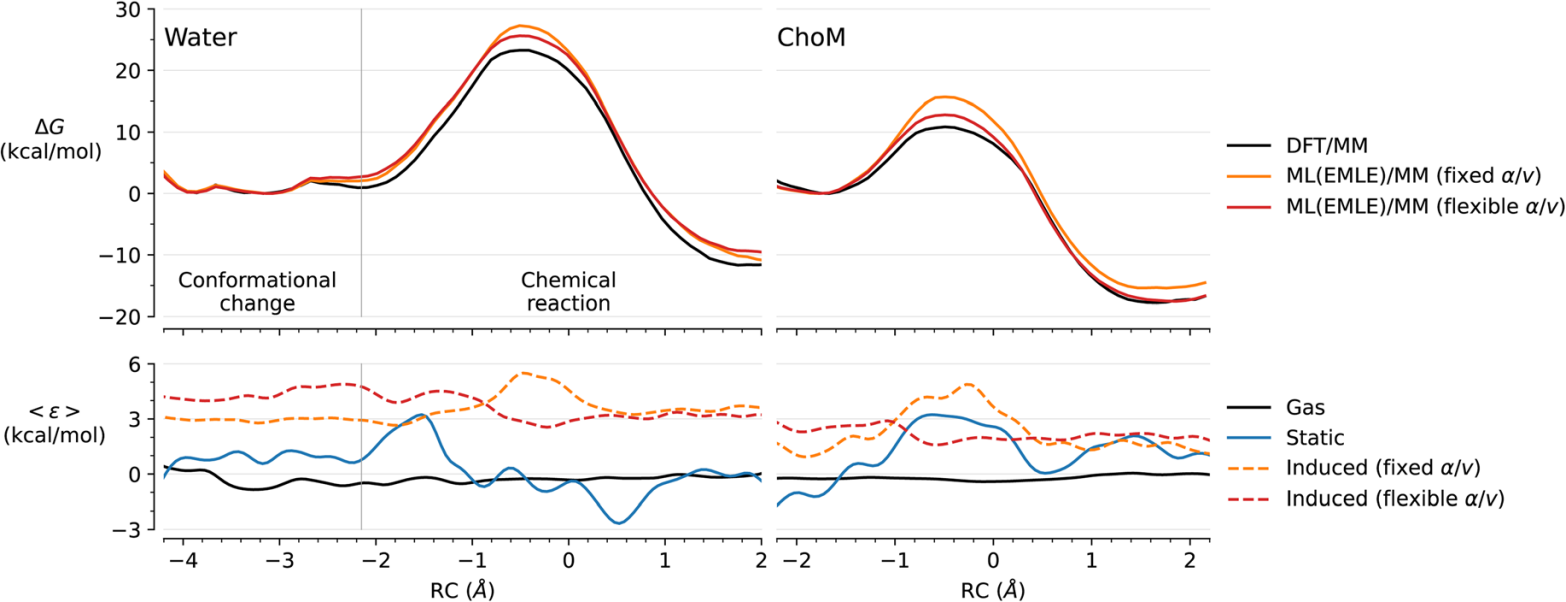
ML/MM simulations of the chorismate to prephenate conversion in water and catalysed by *Ec*ChoM. (Top) Free energy profiles obtained by umbrella sampling for the reference DFT/MM profile and with different ML/MM embedding. All ML/MM simulations share the same gas phase DeepMD MLP. (Bottom) Mean error of the ML(EMLE)/MM potential components. The errors are interpolated using smooth cubic splines interpolation for clarity. The raw values (without interpolation) are provided in Fig. S2.

Free energy profiles for the reaction in water and in enzyme (*Ec*ChoM) were obtained with the reference DFT/MM and with ML/MM with full EMLE embedding using two induction models (Fig. 4) and also compared between different ML/MM embedding models (activation and reaction energies in Table 1). Each ML/MM result is obtained with the same gas phase DeePMD MLP, but different embedding schemes.

**Table 1:** The activation free energies (Δ^‡^*G*), reaction energies (Δ*G*) and catalytic effects (ΔΔ^‡^*G*) for chorismate conversion to prephenate in water solution and catalysed by *Ec*ChoM^a^

	Water		*Ec*ChoM		
	Δ^‡^*G*	Δ_r_*G*	Δ^‡^*G*	Δ_r_*G*	ΔΔ^‡^*G*
B3LYP/6-31G(d)/MM	23.3 ± 1.1	−11.7 ± 0.8	10.8 ± 0.2	−17.8 ± 0.7	−12.5 ± 1.1
ML(EMLE)/MM (flexible *α*/*v*)	25.6 ± 0.8	−9.8 ± 1.1	12.8 ± 0.6	−17.6 ± 0.9	−12.8 ± 1.0
ML(EMLE)/MM (fixed *α*/*v*)	27.3 ± 1.3	−11.0 ± 2.7	15.7 ± 0.3	−15.4 ± 0.5	−11.6 ± 1.3
Mechanical embedding	30.0 ± 1.0	−7.8 ± 0.9	15.4 ± 0.4	−16.3 ± 0.7	−14.6 ± 1.1
MM embedding (*q*_R_)	23.5 ± 1.7	−22.2 ± 1.9	11.9 ± 0.4	−26.8 ± 0.2	−11.6 ± 1.7
MM embedding (*q*_P_)	30.6 ± 1.4	−9.6 ± 1.4	15.2 ± 0.4	−18.1 ± 0.5	−15.4 ± 1.5

a The errors indicate standard deviations obtained from splitting the US windows into 5 fragments and recalculating PMFs.

When comparing the reference DFT/MM results to those from ML/MM, there is a systematic improvement in the predicted results when going from MM embedding to mechanical embedding and to full EMLE embedding. The low errors of the activation energies predicted by MM embedding with chorismate-based charges (*q*_R_) are just a coincidence: the corresponding reaction energies are off by 10.5 kcal mol^−1^ in water and 9.0 kcal mol^−1^ in *Ec*ChoM. Conversely, when prephenate-based MM charges are employed (*q*_P_), errors in reaction energies are much smaller, but this comes at a cost of large errors in the barriers (7.3 kcal mol^−1^ in water and 4.4 kcal mol^−1^ in *Ec*ChoM). This again indicates the inadequacy of fixed charge schemes in ML/MM simulations of chemical reactions, due to poor prediction of (changes in) electrostatic interactions along the reaction. Once the atomic charges respond to the electronic changes in the substrate (mechanical embedding), the results improve significantly, providing more reasonable predictions, with an error range of 2–3 kcal mol^−1^ for reaction energies and 4–6 kcal mol^−1^ for activation free energies.

Inclusion of the EMLE induction term with the fixed *α*/*v* ratio (eqn (5)) reduces the error of the activation energy in aqueous solution (from ∼6 to ∼4 kcal mol^−1^), but not in the enzyme. This is due to the inability of this “fixed” model to describe the change in polarizability of the carbonyl group involved in bond changes. This group becomes more polarizable during the cleavage of the C–O bond, which results in a stronger induced dipole in response to the presence of the nearby positive charge of the catalytic Lys36 (Fig. S3). Incomplete description of this effect leads to weaker TS stabilization and thus to a higher activation energy. When a flexible *α*/*v* ratio (eqn (6)) is used, absolute errors are reduced to 2–2.5 kcal mol^−1^ for the reaction barrier and <2 kcal mol^−1^ for the reaction energy. Notably, the errors in the free energy barrier are systematic, resulting in a prediction of the catalytic effect (difference in barriers between water and enzyme) that is within the statistical error of the PMF calculations performed (≈1 kcal mol^−1^).

It should be noted that the atomic polarizabilities provided by the induction model are not expected to agree with those obtained from the distributed atomic polarizabilities (DAP) approach,^64^ which is based on atoms-in-molecules decomposition of the molecular polarizability tensor into atomic contributions. For instance, the flexible model presented here results in a strongly localized polarization response, assigning large atomic polarizabilities in the TS region only to the atoms directly involved in the chemical process. In contrast, the DAP approach spreads the polarizability over the entire system (Fig. S4, left). Nevertheless, despite such a large conceptual and numeric difference between the two approaches, the molecular isotropic polarizabilities predicted by EMLE are in excellent agreement with the reference QM values (Fig. S4, right). Also, the large assigned atomic polarizabilities do not result in overpolarization, as can be seen from the systematic and positive errors of the induced component across the reaction coordinate (Fig. 4). Since both relevant observables (polarizability and induction energy) are successfully predicted by the “flexible” model, the differences between the predicted atomic *α* values and their DAP counterparts cannot be attributed to a deficiency of the model, as there is not a univocal definition of this property. Whether an induction model trained on DAP data would be more transferable, data efficient, or suitable for a more complete description of response properties (*e.g.* including dispersion or hyperpolarizability), is a matter for future research.

As for AbyU, we calculated the average errors of each of the internal energy components defined in eqn (2)–(4) as a function of the RC (Fig. 4, bottom). Notably, the gas phase error (error of the DeePMD MLP compared to the reference gas phase DFT calculations), is close to zero in both enzyme and water simulations. This confirms that the learning protocol employed for DeePMD converged to an MLP capable of describing the full range of the reaction studied. The quality of the MLP for the conformational change of chorismate taking place before the chemical reaction in aqueous solution is also evidenced by the fact that all tested embedding schemes precisely reproduce the reference PMF in that region (RC from ≈ −4 to −2 Å). This change is dominated by the internal energy of the ML region and therefore is less dependent on the embedding model.

Regarding the remaining energy components, the total error is dominated by the static component of EMLE. To determine whether this error could be reduced by further training, we recalculated the static component using exact charges and valence shell widths from MBIS decomposition of the DFT/MM reference, thus approximating a “perfect” model of the electronic density given the functional form used. Only a minor improvement is seen with such a model (Fig. S2). On the one hand, this indicates that the chosen ML training approach is working well and that the functional capacity of the model is sufficient to reproduce the dependence of the atomic charges on the reaction progress. On the other hand, this means that further improvement of the EMLE scheme would require going beyond the chosen functional form. Since the role of the static model is to predict the gas phase electrostatic potential of the ML region, and both atomic monopoles and charge penetration effects are already taken into account, further improvement can likely be achieved by including higher atomic multipoles (dipoles, quadrupoles *etc.*; see further below).

The systematic positive error of the induced component does not impact the resulting PMF but requires further explanation. It was previously observed that QM/MM tends to overestimate the interaction strength between QM and MM regions for charged^65^ and hydrogen-bonded systems.^66^ This is likely to be especially the case for anionic QM regions, where charge spillover from QM to positive MM point charges may artificially increase the interaction strength. Since EMLE is trained exclusively on gas phase data, it should not suffer from this artifact and can thus underestimate the interaction energy when compared to QM/MM reference energies, resulting in a positive error of the induced component. This hypothesis was confirmed by comparing the radial distribution function (RDF) of water around the substrate from our simulations (with just substrate in QM or ML region) to that obtained when nearby water molecules are also included in the QM region (Fig. S5). The first peak in the ML(EMLE)/MM RDF (indicating the first solvation shell) is at a larger distance than for the DFT/MM RDF, which brings it closer to the RDF obtained when including the first solvation shell into the QM region. The remaining difference between the first RDF peaks at the QM and ML/MM levels may be attributed to the fact that for ML/MM, the short-range repulsion and dispersion is treated at MM level of theory (as standard in QM/MM calculations). Thus, the discrepancy can be reduced either by a bespoke parameterisation of the MM Lennard-Jones parameters or by extending the EMLE scheme to also describe the dispersion and short-range repulsion, as further discussed below.

**Fig. 5:**
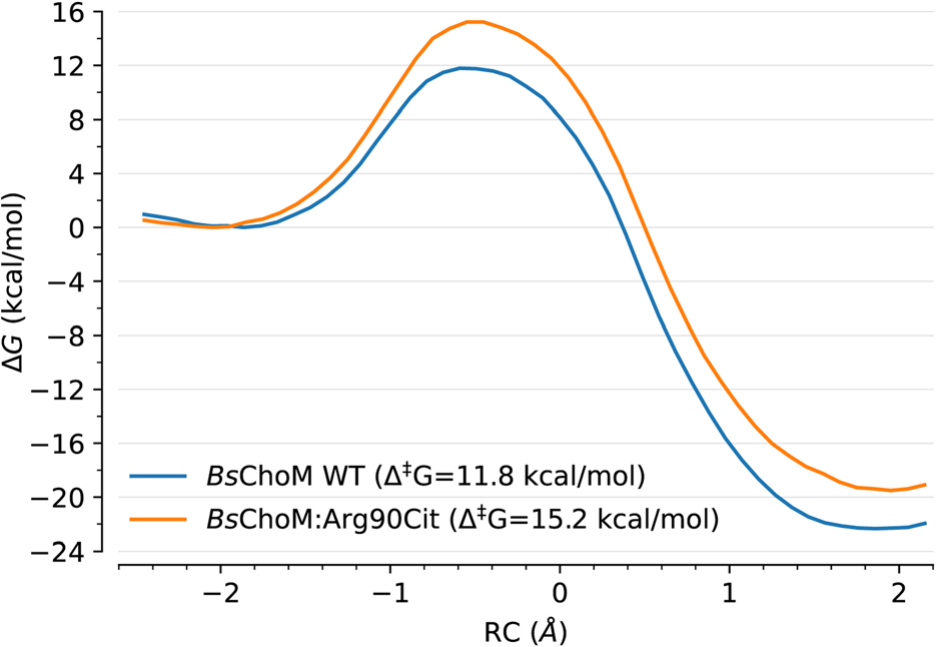
ML(EMLE)/MM free energy profiles of the chorismate to prephenate conversion catalysed by *Bs*ChoM and its Arg90Cit variant. DeePMD and EMLE (flexible *α*/*v*) models as used for the reaction in water and *Ec*ChoM were employed without retraining.

To further show that the ChoM model (MLP and EMLE trained for the chorismate to prephenate reaction) can extrapolate to different environments from those used during the active learning procedure, we have applied it to Chorismate Mutase from *Bacillus subtilis* (*Bs*ChoM), which has a completely different protein fold to *Ec*ChoM, and the deactivated *Bs*ChoM variant where the catalytic arginine (Arg90) is replaced by the isosteric but neutral citrulline (*Bs*ChoM:Arg90Cit). As expected, a model trained exclusively on gas-phase QM reference data successfully extrapolates to a different MM environment: we predict a 3.4 kcal mol^−1^ increase in the free energy barrier of the chemical step upon mutation (Fig. 5), in perfect agreement with the result obtained by Guimarães *et al.*^67^ (3.3 kcal mol^−1^) using QM/MM simulations.

## Discussion

### Current model limitations and possible improvements

The analysis of errors associated with our ML(EMLE)/MM simulations of enzyme reactions indicated that the largest (non-systematic) source of error in both reactions is the static energy. Since *E*_static_ is obtained by predicting the gas phase electrostatic potential of the ML region, the predictions may be significantly improved by expanding the model with static atomic dipoles. MBIS decomposition already provides atomic multipoles of arbitrary order, therefore the training data for such an extension is readily available. In contrast to atomic charges, higher order multipoles are equivariant to the rotation of the molecular system. Prediction of such equivariant properties can be done by switching to equivariant neural networks^19,68–71^ such as those employed in MACE.^19^ This makes further improvement of the EMLE scheme straightforward and will be the topic of future research. On the other hand, simpler and more efficient gas phase MLPs (for example, the DeePMD potential used in the ChoM reaction example) are only able to predict scalar properties. In such cases, extending EMLE with atomic dipoles would require training a separate equivariant model, which will increase the computational cost of both training and inference. In that case, it would be beneficial to adjust the reference atomic charges (obtained from MBIS in this work) to provide better predictions of the electrostatic potential. Another possible strategy to improve the static component of the interaction energy could involve changing the reference atomic charges to better predict the electrostatic potential. It was recently shown that the use of constrained MBIS charges can enhance the description of the electrostatic potential by better predicting higher-order molecular multipoles.^72^ The same result can be achieved by training the static model of EMLE to directly reproduce reference QM/MM *E*_static_ values, while keeping the resulting atomic charges close to MBIS values, as was proposed recently.^73^ Such approaches would directly improve the accuracy of *E*_static_ predictions without requiring explicit atomic dipoles.

While in the case of AbyU, polarization does not have a significant effect in the reaction and the corresponding error of *E*_induced_ is close to zero, the polarizability of the ML region was shown to change dramatically during the conversion of chorismate to prephenate. With a simple, linear relationship between polarizability and atomic volume (as in our initial EMLE approach, fixed *α*/*v* ratio), this resulted in an underestimation of the transition state stabilization (compared to the DFT/MM reference), particularly in the enzyme, and consequently an overestimation of the reaction barrier. By introducing a correction term that takes into account changes in the atomic environment, the description of the induction energy (and thus polarization, flexible *α*/*v* ratio) dramatically improved, resulting in a good agreement between the ML(EMLE)/MM and the reference DFT/MM activation energies and a precise description of the catalytic effect of the enzyme. As discussed above, the remaining systematic positive error in *E*_induced_ observed in the ChoM reaction example is likely due to the limitations of the reference electrostatic embedding QM/MM data. As evidenced by the RDF analysis (Fig. S5), DFT/MM tends to slightly underestimate the distances between the QM region and MM water molecules, thus overpolarizing the QM subsystem, an artifact not observed for ML(EMLE)/MM structures. Since the EMLE model was trained exclusively on gas phase data, it is free of such QM/MM artifacts. This is an important observation for the future development of multiscale potentials. Most of the state-of-the-art QM/MM implementations treat MM atoms as point charges and therefore inevitably suffer from such artifacts. As we show here, the EMLE scheme overcomes this issue by relying on the Thole model based on atomic polarizabilities and thus only includes the environment as the electric field acting on the ML atoms. In addition, errors for systems with significant variations in the electrostatic and response properties (as is the case for the ChoM reaction example presented here) can be mitigated by using larger ML regions, which is feasible due to the low cost of ML/MM simulations. It is expected that the largest errors in both static and induced energy components would arise from the differences in interactions between the reacting groups and the MM environment. Extending the ML region would make these groups farther from the closest MM point charges, reducing significantly the corresponding embedding energy terms.

Another advantage of the EMLE approach is that it allows for the seamless embedding of ML potentials in polarizable MM force fields. While “polarizable QM/MM” methods are actively used in the studies of electronic excitations in condensed phases,^74,75^ they are only rarely employed in reaction simulations and usually require specialized codes. Thanks to a shared mathematical framework, ML(EMLE)/MM could trivially incorporate polarizable force fields based on atomic polarizabilities, such as AMOEBA.^76^

Another possible direction of improvement for ML/MM simulations is the treatment of dispersion and repulsion effects. Both in QM/MM schemes and in the current implementation of EMLE, these are taken into account at the MM level, employing a simple Lennard-Jones potential where parameters remain constant for each atom. Such an approximation is justified in non-reactive simulations, where the chemical nature of atoms is defined in advance. However, it breaks down for chemical processes, where van der Waals interactions of atoms in the reacting species can change dramatically at different stages of the reaction. This deficiency of current QM/MM and many ML/MM potentials can be overcome by including an ML model that would reproduce empirical “dispersion corrections” commonly used in DFT calculations.^77^ Including such a model for QM-MM dispersion/repulsion interactions would considerably improve the description of the processes with significant charge transfer or strong hydrophobic interactions (which is often the case for enzymatic reactions) and is a topic for future research.

### Computational efficiency

The computational efficiency of the ML/MM simulations presented in this work was compared to the reference DFT/MM simulations in two ways. First, we compared the cost of a single energy/forces estimate. This directly measures the efficiency of the ML(EMLE)/MM potential implementation. Second, we compared the amount of sampling achieved over time. This measures the efficiency of the interface with the MD engine. For the two systems presented in this work, the ML/MM energy estimates are at least 2000× faster than their DFT/MM counterparts (Table S3). The performance of ML/MM MD was ≈1000× better than that of DFT/MM. The lower performance boost for MD sampling compared to energy/forces calculations indicates that the *emle-engine* interface overhead is still significant. This is not surprising, since in this work the “external engine” interface of *sander* (from *AmberTools23* (ref. 52)) was employed, which requires using a filesystem for communication between the two codes. The overhead of the interface is expected to reduce significantly if both MD and ML/MM engines are integrated in a single code. Moreover, it should be noted that in both reactions studied here, the ML region was smaller than for a typical QM/MM simulation of enzymatic processes (49 and 24 atoms, respectively). For larger QM (MLP) regions, the boost of employing MLPs is expected to be even more pronounced for two reasons. First, the MLPs usually scale as 
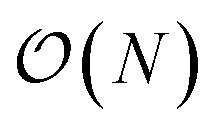
 with the number of atoms, while the scaling of hybrid DFT is 
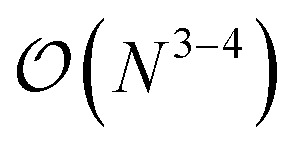
. Second, the MLPs can take advantage of GPU acceleration much better than DFT codes, so a significant increase in the performance boost can be expected for ML regions consisting of >100 atoms.

## Conclusions

Recent advancements in MLPs suitable for reaction modelling allow accurate and efficient reaction simulations, paving the way for computationally efficient protocols that can be incorporated in design workflows. In particular, the coupling of MLPs with MM environments is a promising tool for predictive modelling of biomolecules, such as *de novo* designed or engineered enzymes. In this work, we presented and tested our EMLE ML/MM method for such enzyme reaction simulations. Specifically, we applied our EMLE ML/MM embedding scheme for MLPs to two enzymatic reactions and their counterparts in aqueous solution. The systems were chosen to cover two different aspects of enzymatic catalysis: differences in reactivity of alternative enzyme–substrate conformations and electrostatic stabilization of the transition state (compared to the reactant state) by the enzymatic active site. The compatibility of the EMLE scheme with arbitrary gas phase ML architectures was showcased by choosing MACE and DeepMD to train MLPs for the AbyU and ChoM enzyme reactions, respectively. ML/MM simulations with EMLE provide an accurate description of the reaction in both aqueous solution and in the enzymes, with calculated activation free energies within 2–3 kcal mol^−1^ of the reference DFT/MM values.

Our ML/MM embedding approach allows for the use of MLPs trained in gas phase to be applied to condensed phase processes (such as enzymatic reactions). Importantly, we demonstrate that with the same MLP, the difference in reactivity in response to the environment (in other words, the level of catalysis provided by the enzyme) is captured correctly. This indicates that ML/MM simulations with EMLE will be able to capture the different levels of catalysis provided by different putative enzymes for the same reaction. This would not only apply to enzymes with small differences in sequence, but also for enzymes with significantly different sequences and even different folds, such as those designed by recent AI-based approaches.^3,4^ Therefore, our approach should be suitable for incorporation into modern computational workflows for *de novo* design of biocatalysts. This would allow confident ranking of designs prior to experimental screening, which is often resource intensive. With MLPs trained for a series of putative covalent drugs, similar efficient ML/MM-based activity screening could be applied to enzyme targets (and off-targets) in computational drug design.

## Author contributions

LH, DL, IT, MvdK and KZ designed the study; VG performed all the calculations and analysis of chorismate systems; EC performed all the calculations and analysis of Diels–Alderase systems; MMP performed initial system preparation and simulations for chorismate systems; LH and KZ upgraded *emle-engine* code; RD adjusted ArcaNN code to read ORCA and *emle-server* outputs; KZ and IT supervised VG; MvdK supervised EC; KZ, MdlP and DL supervised MMP; MvdK and KZ coordinated the project; MvdK, IT and KZ obtained funding; VG, EC, IT, MvdK and KZ prepared the manuscript.

## Conflicts of interest

There are no conflicts to declare.

## Abbreviations

EMLE: Electrostatic machine learning embedding
GPU: Graphics processing unit
MD: Molecular dynamics
ML/MM: Machine learning potential/molecular mechanics
MLPs: Machine-learned potentials
MM: Molecular mechanics
PDB ID: Protein data bank identifier
PMF: Potential of mean force
QM: Quantum mechanics
QM/MM: Quantum mechanics/molecular mechanics
RC: Reaction coordinate
RDF: Radial distribution function
US: Umbrella sampling
WHAM: Weighted histogram analysis method

## Supplementary Material

SC-017-D6SC01156J-s001

## Data Availability

Simulation files, MLP and EMLE models trained, as well as raw data and Jupyter notebooks used to generate all the figures in this publication, are provided in the supporting GitHub repository (https://github.com/chemle/emle-enzymes-paper). Supplementary information (SI): Fig. S1: version of Fig. 2 with the error analysis plotted without smoothing, and errors for an updated MACE MLP; Fig. S2: version of Fig. 4 without error smoothing and including the static component error based on exact MBIS atomic properties; Fig. S3: variation of atomic polarizabilities with fixed and flexible *α*/*v* ratios; Fig. S4: comparison of atomic and molecular polarizabilities from DAP and EMLE; Fig. S5: the radial distribution functions of water oxygen atoms with respect to all the atoms of chorismate. Table S1: free energies and errors for the AbyU reaction simulations; Table S2: convergence analysis of AbyU ML(EMLE)/MM US simulations; Table S3: performance comparison between ML/MM and reference DFT/MM calculations. See DOI: https://doi.org/10.1039/d6sc01156j.
