# Supplementary material for: Simulating enzyme catalysis with electrostatically embedded machine learning potentials
Source: Chem Sci. 2026 Mar 10;17(17):8542–56. doi: 10.1039/d6sc01156j (PMC12974892; doi:10.1039/d6sc01156j)
Supplement: SC-017-D6SC01156J-s001 [file SC-017-D6SC01156J-s001.pdf]

Supplementary Information (SI) for Chemical Science.
This journal is © The Royal Society of Chemistry 2026

# Simulating enzyme catalysis with electrostatically embedded machine learning potentials

## *Supplementary Information*

*Valentin Gradisteanu[**,1], Elliot W. Chan[**,2], Lester Hedges[2,3], Meritxell Malagarriga[1], Rolf David[4], Miguel de la Puente[4], Damien Laage[4], Iñaki Tuñón[1], Marc W. van der Kamp[*,2], Kirill Zinovjev[*,1]*

[1] Departamento de Química Física, Universidad de Valencia, 46100, Burjassot (Spain)

[2] School of Biochemistry & Cellular and Molecular Medicine, University of Bristol, Biomedical Sciences Building, University Walk, Bristol BS8 1TD, UK

[3] OpenBioSim Community Interest Company, Edinburgh, United Kingdom

[4] Laboratory CPCV, Department of Chemistry, École Normale Supérieure, PSL University, Sorbonne Université, CNRS, Paris (France)

*to whom correspondence should be addressed: marc.vanderkamp@bristol.ac.uk; kirill.zinovjev@uv.es

**both authors contributed equally

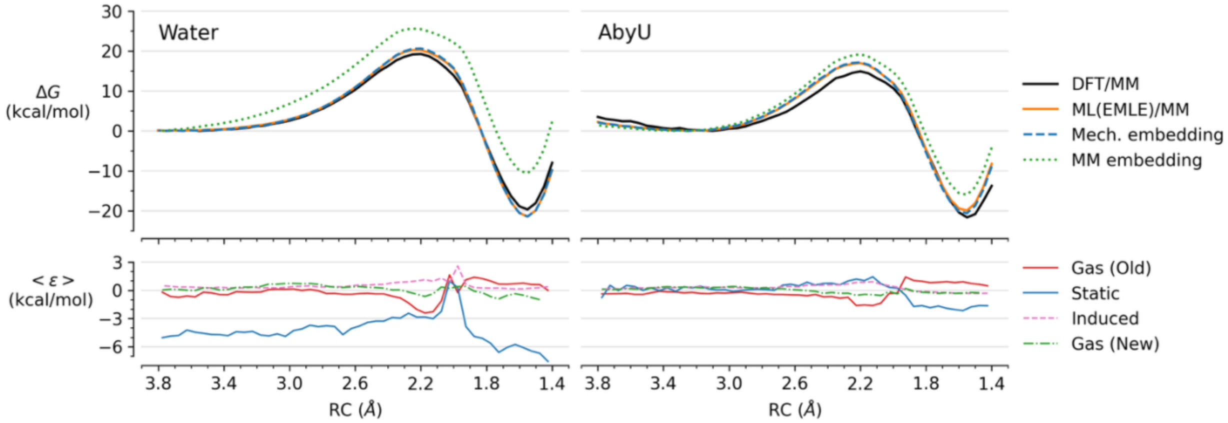


**Figure S1**. Version of Figure 2 ("ML/MM simulations of the Diels-Alder reaction for the AbyU substrate in water and catalysed by AbyU"), with the error analysis plotted without smoothing across the windows with cubic splines. 'Gas (New)' indicates the errors (vs. DFT) calculated on the same conformations, but energies calculated with an updated MACE MLP, resulting in errors <1.0 kcal/mol in both systems. The updated MLP was obtained by adding 60 more structures to the training set, 5 each from the umbrella sampling windows between RC=2.4 (just before the transition state) and RC=1.3 (product).

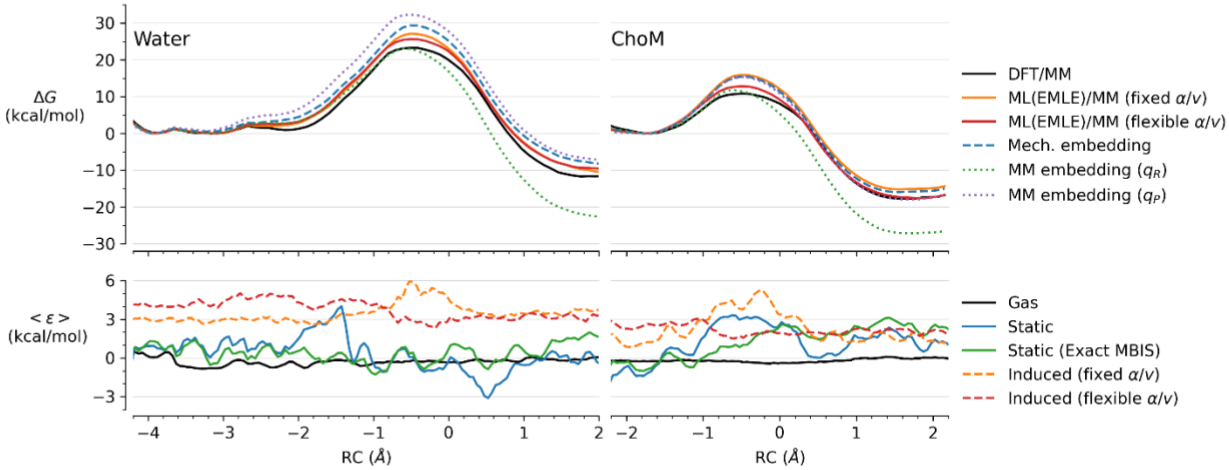


**Figure S2**. Version of Figure 4 ("ML/MM simulations of the chorismate to prephenate conversion in water and catalysed by ChoM") with the error analysis plotted without smoothing across the windows with cubic splines. The static component error based on exact MBIS atomic properties derived from DFT (atomic core/valence charges and shell widths) is also plotted.

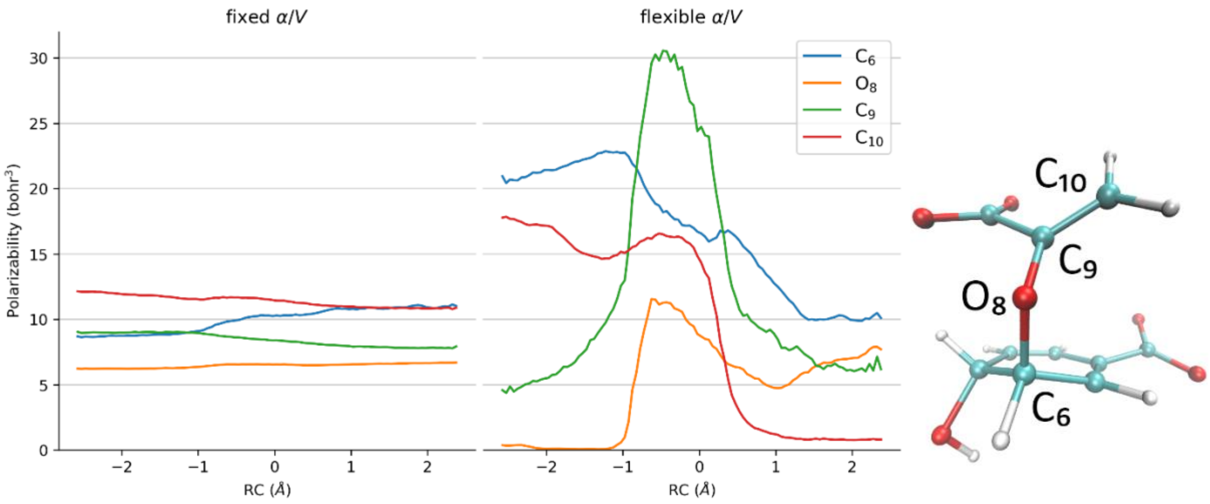


**Figure S3**. Atomic polarizabilities with the largest variations in the "flexible" polarizability model (Main text, Eq. 6) during the chorismate to prephenate reaction.

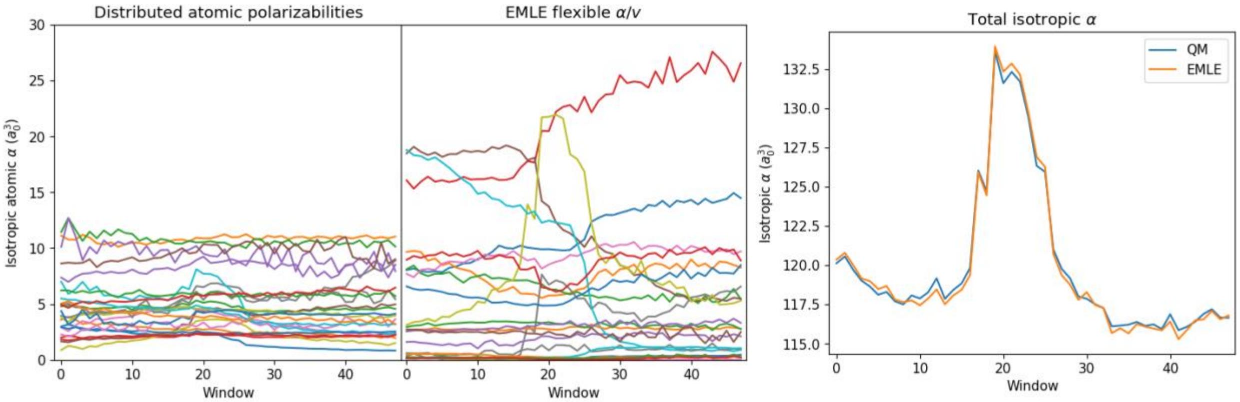


**Figure S4**. Left: isotropic atomic polarizabilities obtained with distributed atomic polarizabilities analysis (as implemented in ORCA 6.1) and from Thole model implementation in EMLE. Right: isotropic molecular polarizabilities from reference DFT calculations and predicted by EMLE.

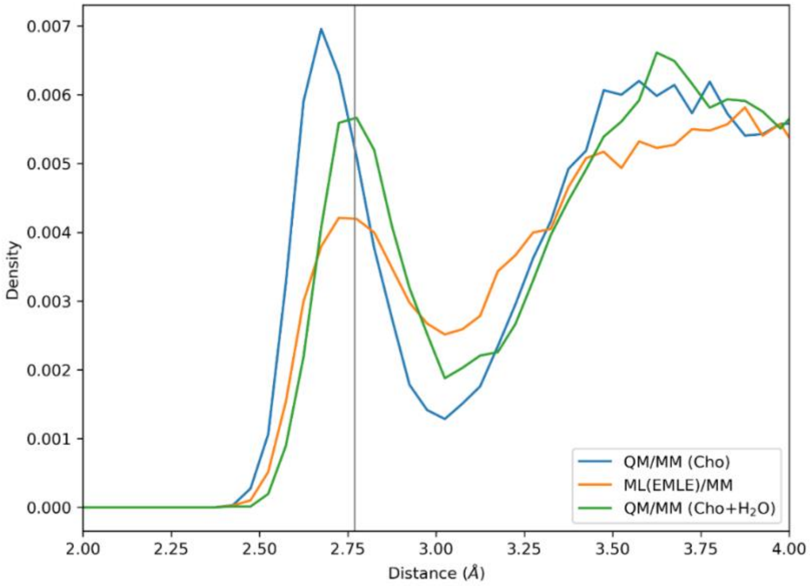


**Figure S5**. The radial distribution functions of water oxygen atoms with respect to all the atoms of chorismate. Blue: QM/MM with chorismate in the QM region; Yellow: ML(EMLE)/MM with chorismate treated with ML; green: QM/MM with chorismate and first solvation shell in the QM region.

**Table S1**. The activation free energies ($\Delta^{\ddagger}G$) and reaction energies ($\Delta G$) for Diels-Alder reaction in water solution and catalysed by AbyU. The errors are calculated by splitting the US windows into 5 fragments and calculating standard deviations of obtained barriers and reaction energies.

| | **Water** | | **Pose 1** | | **Pose 2** | |
|---|---|---|---|---|---|---|
| | $\Delta^{\ddagger}G$ | $\Delta_rG$ | $\Delta^{\ddagger}G$ | $\Delta_rG$ | $\Delta^{\ddagger}G$ | $\Delta_rG$ |
| **M06-2X/6-31G(d)/MM** | 19.3 ± 0.5 | –19.7 ± 1.2 | 14.9 ± 0.6 | –21.7 ± 1.1 | 22.3 ± 0.9 | –19.1 ± 3.5 |
| **ML(EMLE)/MM** | 20.2 ± 0.2 | –21.5 ± 0.7 | 17.0 ± 0.8 | –19.9 ± 0.8 | 22.9 ± 0.9 | –15.1 ± 1.5 |
| **Mechanical embedding** | 20.6 ± 0.3 | –21.5 ± 1.0 | 17.2 ± 0.6 | –20.6 ± 1.4 | 22.3 ± 1.1 | –15.7 ± 2.2 |
| **MM embedding** | 25.6 ± 0.4 | –10.6 ± 3.2 | 19.2 ± 0.7 | –16.0 ± 1.7 | 21.3 ± 0.8 | –18.8 ± 3.3 |

**Table S2**. Convergence analysis for the activation free energies ($\Delta^{\ddagger}G$) and reaction energies ($\Delta G$) for the Diels-Alder reaction catalysed by AbyU (pose 1), as calculated from the ML(EMLE)/MM umbrella sampling simulations. Note: sampling is performed for 5 different initial positions, hence a period of 0-1 ps indicates 5 ps of sampling per window.

| | | Pose 1 | | Difference from 0-10 ps | |
|---|---|---|---|---|---|
| **Time interval (ps)** | Total sampling per window (ps) | $\Delta^{\ddagger}G$ | $\Delta_r G$ | $\Delta^{\ddagger}G$ | $\Delta_r G$ |
| **0-1** | 5 | 17.52 | -19.47 | 0.540 | 0.464 |
| **0-2** | 10 | 17.39 | -18.73 | 0.409 | 1.199 |
| **0-3** | 15 | 17.29 | -18.94 | 0.308 | 0.989 |
| **0-4** | 20 | 17.17 | -19.27 | 0.193 | 0.659 |
| **0-5** | 25 | 17.01 | -19.59 | 0.032 | 0.346 |
| **5-10** | 25 | 16.94 | -20.33 | -0.038 | -0.397 |
| **0-10** | 50 | 16.98 | -19.93 | n/a | n/a |

**Table S3**. Performance comparison between ML/MM and reference DFT/MM calculations. All calculations were performed on a single core of an Intel Xeon Platinum 8480+ 56C 2GHz processor.

| | **AbyU** | | **ChoM** | |
|---|---|---|---|---|
| | Forces (s) | MD (ps/day) | Forces (s) | MD (ps/day) |
| DFT/MM | 773 | 0.112 | 242 | 0.356 |
| ML(EMLE)/MM | 0.326 | 137 | 0.0520 | 325 |
| **Boost** | **2371** | **1223** | **4654** | **913** |
